# Combination of bazedoxifene with chemotherapy and SMAC-mimetics for the treatment of colorectal cancer

**DOI:** 10.1038/s41419-024-06631-8

**Published:** 2024-04-10

**Authors:** Rhynelle S. Dmello, Michelle Palmieri, Pathum S. Thilakasiri, Larissa Doughty, Tracy L. Nero, Ashleigh R. Poh, Sarah Q. To, Erinna F. Lee, W. Douglas Fairlie, Lisa Mielke, Michael W. Parker, Ivan K. H. Poon, Eduard Batlle, Matthias Ernst, Ashwini L. Chand

**Affiliations:** 1 Olivia Newton-John Cancer Research Institute and School of Cancer Medicine, La Trobe University, Heidelberg, VIC 3084 Australia; 2 Walter and Eliza Hall Institute of Medical Research (WEHI), Parkville, VIC 3010 Australia; 3 Department of Biochemistry and Pharmacology, and ACRF Facility for Innovative Cancer Drug Discovery, Bio21 Molecular Science and Biotechnology Institute, The University of Melbourne, Parkville, VIC 3010 Australia; 4 Department of Biochemistry and Chemistry, School of Agriculture, Biomedicine and Environment, La Trobe Institute for Molecular Science, La Trobe University, Bundoora, VIC 3083 Australia; 5 ACRF Rational Drug Discovery Centre, St. Vincent’s Institute of Medical Research, Fitzroy, VIC 3065 Australia; 6 Institute for Research in Biomedicine (IRB Barcelona), The Barcelona Institute of Science and Technology (BIST), Barcelona, Spain; 7 Centro de Investigación Biomédica en Red de Cáncer (CIBERONC), Barcelona, Spain; 8 Institució Catalana de Recerca i Estudis Avançats (ICREA), Barcelona, Spain

**Keywords:** Targeted therapies, Apoptosis, Colorectal cancer, Chemotherapy

## Abstract

Excessive STAT3 signalling via gp130, the shared receptor subunit for IL-6 and IL-11, contributes to disease progression and poor survival outcomes in patients with colorectal cancer. Here, we provide evidence that bazedoxifene inhibits tumour growth via direct interaction with the gp130 receptor to suppress IL-6 and IL-11-mediated STAT3 signalling. Additionally, bazedoxifene combined with chemotherapy synergistically reduced cell proliferation and induced apoptosis in patient-derived colon cancer organoids. We elucidated that the primary mechanism of anti-tumour activity conferred by bazedoxifene treatment occurs via pro-apoptotic responses in tumour cells. Co-treatment with bazedoxifene and the SMAC-mimetics, LCL161 or Birinapant, that target the IAP family of proteins, demonstrated increased apoptosis and reduced proliferation in colorectal cancer cells. Our findings provide evidence that bazedoxifene treatment could be combined with SMAC-mimetics and chemotherapy to enhance tumour cell apoptosis in colorectal cancer, where gp130 receptor signalling promotes tumour growth and progression.

## Introduction

Inflammation represents one of the hallmarks of cancer [1]. It plays a crucial role in colorectal tumour initiation and progression [2] by contributing to tumour cell proliferation, epithelial-to-mesenchymal transition (EMT), stemness [3] and immune evasion [4]. Significant effort has been undertaken to identify upstream activators of Signal Transducer and Activator of Transcription 3 (STAT3) and how these factors can be exploited to develop new therapies for colorectal cancer [5, 6]. Notably, the interleukin-6 (IL-6) family of cytokines, including IL-6, IL-11 and leukaemia inhibitory factor (LIF), directly activate STAT3 and contribute to the establishment and progression of colorectal cancer [7–10].

Increased STAT3 activation, as assessed by the presence of the tyrosine phosphorylation isoform of the protein, is observed in more than 50% of colorectal cancer tumours [11]. This correlates with a higher incidence of lymph node metastasis and poorer patient prognosis [12]. Excessive STAT3 activity is associated with higher mortality rates and peritumoral (Crohn’s-like) lymphocytic structures, implicating a role for STAT3 in regulating immune-mediated tumour responses [11]. However, very little about the STAT3-dependent cellular mechanisms underpinning colorectal cancer progression is understood. It remains unclear whether pharmacological inhibition of STAT3 activity, in combination with standard-of-care chemotherapy, could enhance cancer cell death.

Drugs that inhibit upstream activators of STAT3, i.e. inhibitors of the IL-6 family of cytokines, represent a promising therapeutic strategy to curb tumour growth [6]. These include tocilizumab, a monoclonal anti-IL-6 antibody that prevents the binding of IL-6 to its cognate receptor subunit IL-6R and subsequent complex formation with the transmembrane gp130 receptor subunit. IL-6 signals via a hexameric protein complex comprised of IL-6:IL-6R:gp130 in a ratio of 2:2:2, where IL-6R is the cytokine-specific receptor subunit, and gp130 is the receptor subunit shared by IL-6 cytokine family members. Tocilizumab has been trialled as a therapy in various inflammatory conditions such as Castleman’s and Crohn’s diseases; and chronic lymphocytic leukaemia, breast, colorectal and ovarian cancers [6]. Currently, no approved therapeutics specifically block either LIF or IL-11-mediated signalling. However, preclinical studies suggest that targeting these cytokines may reduce cancer growth [13–15].

The United States Food and Drug Administration (FDA) approved drug bazedoxifene (BZA) was initially designed as a selective estrogen receptor modulator. Recently, BZA has been shown to block IL-6 and IL-11-dependent STAT3 activation [5, 16, 17]. We have previously demonstrated that BZA treatment significantly reduced gastric and colorectal cancer development and progression in preclinical animal models [5]. Importantly, we showed that BZA is the first drug to concurrently inhibit both IL-6 and IL-11 signalling and suppress STAT3 activation in cancer cells [5].

In the current study, we demonstrate that BZA binds directly to gp130 and has clinical utility as an anti-cancer agent using a range of human colorectal cancer models. Our data demonstrated that BZA potently suppressed the growth of human colorectal cancer xenografts. The proliferation of patient-derived organoids was significantly reduced by BZA treatment. Our data identified the activation of apoptotic pathways as the primary mechanism via which BZA treatment reduces the growth of colorectal cancer. Interestingly, there was an increase in the expression of the Inhibitor of Apoptosis Proteins (IAP) family of proteins (c-IAP, XIAP and SMAC) observed with BZA treatment. Therefore, we explored whether combining BZA with SMAC-mimetics could improve apoptosis rates in colorectal cancer cells. Co-treatment of BZA with either LCL161 or Birinapant significantly increased apoptosis rates and decreased proliferation rates, identifying a novel and effective treatment combination for colorectal cancer.

Our findings suggest that BZA can be used as an effective adjuvant to boost the efficacy of chemotherapies. The most significant apoptosis rate in patient-derived organoids was observed when BZA was combined with the topoisomerase inhibitor SN38. This work supports the rationale that targeting gp130-dependent STAT3 signalling via the dual inhibition of IL-6 and IL-11 is a novel colorectal cancer treatment opportunity.

## Results

### BZA interacts with gp130 and suppresses STAT3 activation

**Fig. 1:**
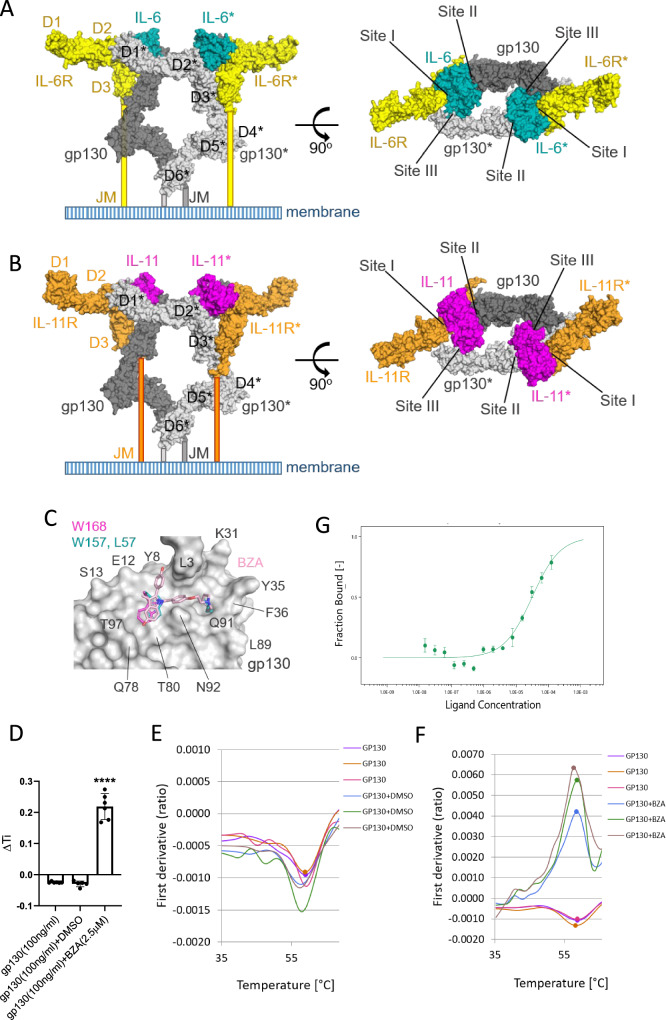
BZA interacts with gp130 ECD at Site III. **A** A model of the IL-6 receptor ECD was constructed (as described in the Methods), the hexameric protein complex comprises two IL-6:IL-6R:gp130 trimers interacting via two Site III interfaces. An asterisk denotes the components of one trimer. The individual proteins are depicted as molecular surfaces: IL-6 (blue), IL-6R (yellow) and gp130 (grey). Two orthogonal views are shown; the view on the right looks down onto the membrane. **B** A model of the IL-11 receptor ECD was constructed in a similar manner. The individual proteins are shown as molecular surfaces: IL-11 (magenta), IL-11R (orange) and gp130 (grey). **C** Putative binding mode of BZA (light pink sticks) to Site III located on gp130 D1 (grey molecular surface). BZA can mimic the interactions of IL-6 residues L57 and W157 (blue sticks), as well as the interaction of IL-11 W168 (magenta sticks), with Site III residues located on gp130 D1 [5]. **D** Change in the inflection temperature (∆Ti) of gp130 ECD alone, and incubated with either DMSO or 2.5 µM BZA for 5 min at RT. Error bars are mean ± SD of *n* = 6 independent experiments. Student’s unpaired t-test: ****p < 0.0001. **E** Thermal shift profile of human recombinant gp130 ECD after incubation with vehicle DMSO for 5 min at RT of *n* = 3 independent experiments. Each line denotes a separate investigation. **F** Thermal shift profile of gp130 ECD after incubation with 2.5 µM BZA for 5 min at RT of *n* = 3 independent experiments. Each line denotes a separate experiment. **G** Quantification of the binding interaction between gp130 ECD and BZA using microscale thermophoresis (MST). The binding curve for 40 nM labelled gp130 with a titration series of BZA. The *K*_D_ of BZA binding to gp130 ECD was determined to be 33.7 ± 8.2 µM (*n* = 2 independent replicates, error bars represent mean ± SD).

We have previously shown that BZA interferes with IL-6 and IL-11 signalling to suppress tumour growth in preclinical models of gastric and colon cancer [5]. The signalling complexes for IL-6 and IL-11 are thought to assemble in a stepwise manner, as illustrated for IL-11 in Fig. S1A. All eleven cytokines in the IL-6 family interact with their specific receptor subunit via Site I, and with gp130 via Site II, to form trimeric complexes (Figs. S1A, 1A-B). However, only IL-6 and IL-11 signal via a hexameric receptor complex that is comprised of two of the aforementioned trimers, thereby forming the Site III interaction surface between the cytokine and gp130 of the second receptor trimer. Site III is a unique interaction surface for these two cytokines (Fig. 1A, B). BZA mimics the interactions made by IL-6 residues L57 and W157 (shown as blue sticks in Fig. 1C), or by IL-11 residue W168 (shown as magenta sticks in Fig. 1C), with the Site III residues in domain D1 of the extracellular domain (ECD) of gp130. Thus, when BZA binds to Site III of the D1 domain in gp130 it blocks the dimerisation of the two trimeric receptor complexes, thereby preventing the formation of the signalling-competent hexameric complex (Fig. S1A).To substantiate our previous observations (Fig. S1B and [5]), we first assessed the direct interaction of BZA with the human gp130 ECD.

We evaluated the interaction between BZA and the gp130 ECD by measuring changes in the intrinsic fluorescence of tryptophan and tyrosine residues that result from alterations to the 3D structure of proteins with increasing temperature. Tycho (Nanotemper) is a thermal ramping instrument that detects changes in fluorescence at 350 and 330 nm, measuring the transition from folded to unfolded with increasing temperature. The temperature at which an unfolding transition occurs is called the inflection temperature (Ti). A change in Ti was observed, from −0.02966 (mean) for the gp130 protein alone to a Ti of 0.20766 (mean) when the gp130 ECD was incubated with BZA (Fig. 1D). We also observed an increase in the 350/330 nm ratio of fluorescent intensity from between −0.001 and −0.0015 for the gp130 protein (Fig. 1E) to between 0.004 and 0.006 in the presence of BZA (Fig. 1F). The thermal shift, from a minima to a maxima, indicated a significant conformational stabilisation of the gp130 ECD upon co-incubation with BZA. These changes in the gp130 ECD thermal profile upon incubation with BZA indicated increased thermal stability of the protein, confirming that BZA was binding directly to the gp130 ECD.

We next used microscale thermophoresis (MST) to estimate the binding affinity (*K*_D_) of gp130 ECD and BZA. MST is a solution-based technique where fluorescently-labelled gp130 ECD is free to move in solution along a thermal gradient. Temperature-induced changes in movement due to the concentration of bound BZA are quantified. The gp130 ECD bound BZA with an estimated *K*_D_ of 33.7 ± 8.2 µM (Fig. 1D), which is consistent with the reported *K*_D_ range 140–180 µM obtained using an orthogonal biophysical technique, surface plasmon resonance (SPR) [18, 19].

Finally, we used a STAT3-responsive, APRE-luciferase reporter assay to demonstrate that the induction of STAT3 activity resulting from IL-6 and IL-11 treatment was significantly inhibited with co-treatment of BZA (Fig. S1C-D).

### Reduction of gp130 receptor activity inhibits the growth of colorectal cancer xenografts

**Fig. 2:**
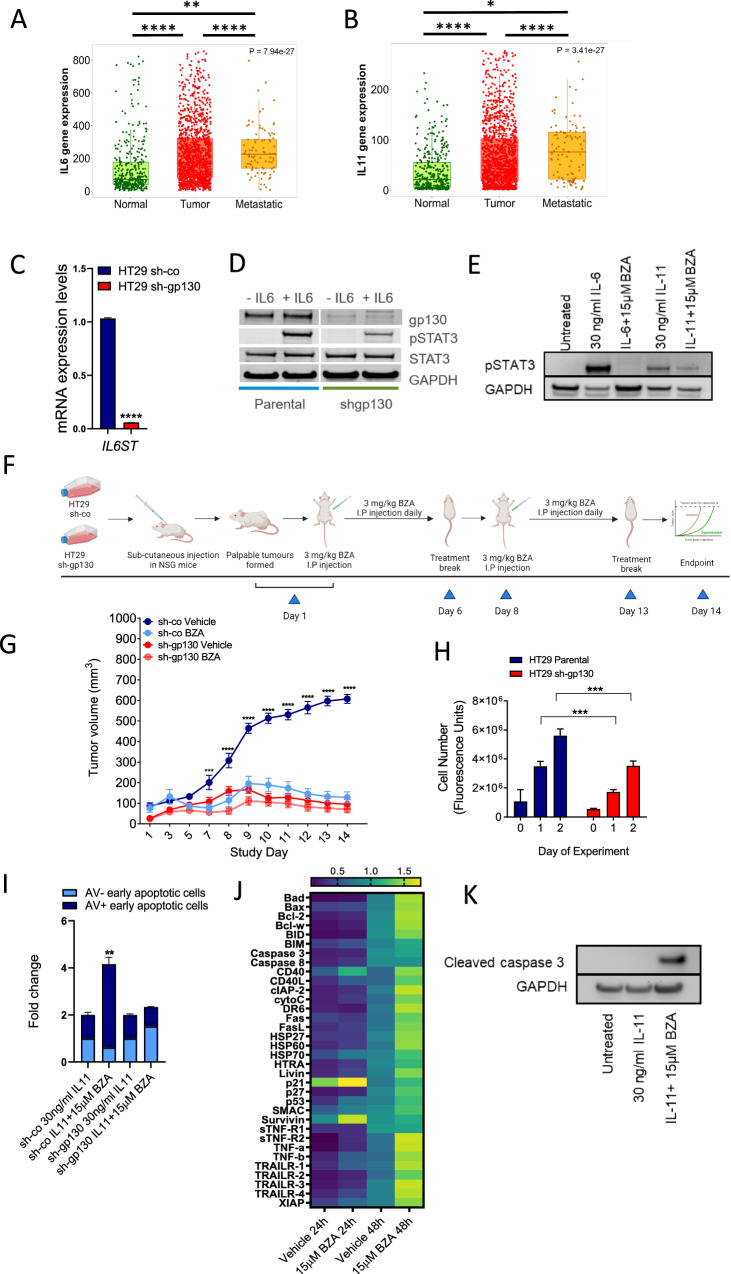
BZA activity potently suppresses the growth of human colorectal cancer xenografts in vivo. Gene expression analysis of *IL6* and (**A**) and *IL11* (**B**) in normal patients (*n* = 377), patients with primary colon tumours (*n* = 1450) and patients with metastatic disease (*n* = 99) taken from The Cancer Genome Atlas (TCGA) database. Dunnett’s multiple comparisons test: ****p < 0.0001, ***p < 0.001, *p < 0.05. **C** mRNA expression analysis (∆∆Ct values) of gp130 (*IL6ST)* compared to the housekeeping gene *GAPDH* in HT29 sh-co and HT29 sh-gp130 cells. Error bars are mean ± SEM of *n* = 3 replicates. Student’s unpaired t-test: ****p < 0.0001. **D** Western blot analysis of gp130 and pSTAT3 expression in HT29 sh-co (short hairpin control) and HT29 sh-gp130 (short hairpin gp130 knockdown) cells following stimulation with 30 ng/ml of IL-6 for 15 min. **E** Western blot of pSTAT3 protein expression in HT29 sh-co cells after treatment with 15 µM BZA in the presence of 30 ng/ml IL-6 and IL-11. **F** Schematic representation of HT29 sh-co and sh-gp130 treatment with BZA in vivo. Treatment was initiated in all experimental cohorts when HT29 sh-co xenografts reached 100 mm^3^ (shown as Day 1 in Fig. 2G). **G** Tumour volumes of HT29 sh-co and HT29 sh-gp130 xenograft tumours treated with 3 mg/kg of BZA over 14 days. Error bars are mean ± SEM of *n* = 5 mice per group. A comparison of mean tumour volumes across experimental groups was performed with Student’s unpaired t-test. P values are shown as ****p < 0.0001 and ***p < 0.001. All statistically significant values are indicated on the graph. Data points on the graph that are not statistically significant are unlabelled. **H** Quantification of cell proliferation in HT29 sh-co and HT29 sh-gp130 cells over 2 days in vitro. Error bars are mean ± SEM of *n* = 3 replicates. One-way ANOVA with Tukey’s multiple comparisons tests: ***p < 0.001. **I** Flow cytometry analysis of Annexin V-FITC (AV) and TO-PRO-3-iodide positive HT29 sh-co (short hairpin control) and HT29 sh-gp130 (short hairpin gp130) cells following treatment with 15 μM BZA in the presence of 30 ng/ml of IL-11 for 48 hours. Error bars are mean ± SD of *n* = 2 independent experiments. Fold change relative to untreated controls (without IL-11). Student’s unpaired t-test: **p < 0.01. **J** Apoptotic pathway protein expression was analysed using a dot antibody array (in HT29 sh-co cells treated with 15 μM BZA in the presence of 30 ng/ml of IL-11 for 24 hours and 48 hours. Protein expression is relative to positive antibody control. Data is the mean ± SD of *n* = 2 replicates. Statistical analysis was conducted using a two way ANOVA with Tukeys multiple comparisons test. P-values are indicated in Supplementary Table 2. **K** Western blot of cleaved caspase 3 expression in HT29 sh-co cells after treatment with 15 µM BZA in the presence of 30 ng/ml IL-11 for 48 hours.

We first analysed *IL6* and *IL11* gene expression from human transcriptomics datasets in The Cancer Genome Atlas (TCGA). We found *IL6* (Fig. 2A), and *IL11* (Fig. 2B) expression was significantly upregulated in patients with primary tumours (*n* = 1450), as well as in patients with metastatic lesions (*n* = 99) when compared to expression levels in the normal colon of patients (*n* = 377). This suggests that therapeutic inhibition of IL-6 and IL-11 signalling via inhibition of the gp130 receptor could be a beneficial treatment option for colorectal cancer patients. To evaluate whether suppression of gp130-mediated signalling inhibited the growth of human colorectal cancer cells, we used short hairpin RNA to suppress gp130 expression in the colorectal cancer cell line, HT29 (referred to as HT29 sh-gp130) [20]. HT29 is a microsatellite stable (MSS) and highly metastatic human colorectal cancer cell line characterised by BRAF V600E, p53 R273H (gain of function) and PIK3CA P449T mutations. BZA-treatment naïve HT29 sh-gp130 cells showed decreased expression of the *IL6ST* transcript encoding gp130 (Fig. 2C), reduced gp130 protein expression (Figs. 2D and S6A) and reduced STAT3 activation when compared to expression levels in the HT29 shRNA-control transfected cells (referred to as HT29 sh-co) (Figs. 2D and S6A). mRNA expression levels of *IL11* and *IL11RA* were also assessed to confirm the presence of active signalling components in HT29 sh-co and sh-gp130 cells (Fig.S2A). As BZA is a selective estrogen receptor modulator (SERM), the mRNA transcript levels of estrogen receptor α (*ESR1*) and β (*ESR2*) were also evaluated in the HT29 cells and found to be minimally expressed compared to the expression *of IL11* and *IL11RA* (Fig. S2A).

We next evaluated whether BZA treatment inhibited gp130-dependent signalling in vitro by assessing protein expression of pSTAT3 after treatment of HT29 sh-co cells with 15 µM BZA for 30 min (Figs. 2E and S6B). The HT29 sh-co cells resulted in increased phosphorylation of STAT3 when stimulated with 30 ng/ml of IL-6 and IL-11 for 15 min (Figs. 2E and S6B). This activation of STAT3 was greatly reduced with 15 µM BZA in the IL-6 and IL-11 treated cells (Figs. 2E and S6B). We then evaluated whether BZA treatment reduced tumour burden in vivo by subcutaneously engrafting HT29 sh-co and HT29 sh-gp130 cells into immune-compromised NOD scid gamma (NSG) mice (Fig. 2F). Following the detection of palpable tumours (100 mm^3^), mice were treated with 3 mg/kg of BZA five times a week (Fig. 2F). We observed significantly reduced tumour growth in the animals harbouring HT29 sh-co xenografts when treated with 3 mg/kg of BZA compared to the vehicle treatment cohort (Fig. 2G). No toxicity was observed in our studies with 3 mg/kg of BZA treatment [5, 21, 22]. HT29 sh-gp130 xenografts established and grew significantly slower compared to HT29 sh-co xenografts showing a dependence of tumours to gp130/STAT3 signalling activity (Fig. 2G, H). The treatment of HT29, sh-gp130 xenografts with BZA did not further suppress tumour growth (Figs. 2G and S2B-C). In addition, the knockdown of gp130 in HT29 cells reduced cell proliferation rates in vitro compared to HT29 sh-co cells (Fig. 2H).

We wanted to establish whether the impaired growth observed in BZA-treated animals could also be attributed to increased apoptosis of tumour cells. We exposed HT29 sh-co and HT29 sh-gp130 cells to BZA in vitro for 48 hours in serum-free media supplemented with IL-11. To measure cells in different stages of apoptosis we used flow cytometry to quantify levels of Annexin V and TO-PRO-3 staining. Flow cytometry plots for each treatment showing distinct Annexin V + TO-PRO-3+ early apoptotic cells, Annexin V- TO-PRO-3+ early apoptotic cells, viable cells and necrotic cells are shown in Fig. S2D. Flow cytometry gating to differentiate between Annexin V + TO-PRO-3+ early apoptotic cells, Annexin V- TO-PRO-3+ early apoptotic cells and necrotic cells was performed according to [23] and is shown in our model in Fig. S3A. We observed increased Annexin V and TO-PR-O3 staining (indicative of early-stage apoptosis) in HT29 sh-co cells compared to HT29 sh-gp130 controls (Fig. 2I). Treatment of HT29 cells with BZA for either 24 hours or 48 hours induced expression of cleaved caspases 3 and 8 (full-length pro-form and activated form), pro-apoptotic proteins of the Bcl-2 family (Bad, Bax, Bid and Bim), members of the TNF-α family (TRAIL-1 and 2, TNF-α, CD40 and Fas), p53, the inhibitor of apoptosis (IAP) family of proteins (c-IAP, XIAP, SMAC and HTRA) and heat shock proteins (HSP-27, HSP-60 and HSP-70) using protein arrays (Fig. 2J). We confirmed that protein expression of cleaved caspase 3 was significantly increased on treatment with BZA in the presence of IL-11 after 48 hours using Western blot analysis (Figs. 2K and S6C).

Collectively, our results demonstrate that sh-RNA mediated knockdown or pharmacological inhibition of gp130-mediated signalling with BZA treatment effectively reduced tumour burden or induced tumour cell apoptosis.

### Inhibition of gp130/STAT3 signalling initiates pro-apoptotic responses in colorectal cancer cells

**Fig. 3:**
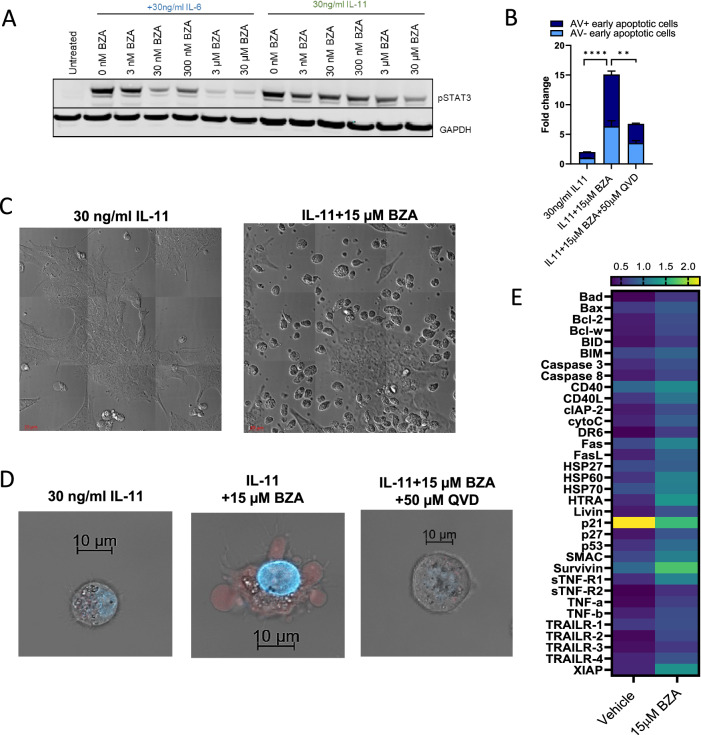
Inhibition of gp130/STAT3 signalling initiates pro-apoptotic responses in colorectal cancer. **A** Western blot of pSTAT3 and GAPDH protein expression levels in LIM2405 cells treated with BZA (3 nM - 30 µM) for 1 hour and stimulated with 30 ng/ml IL-6 and IL-11 for 15 min. **B** Flow cytometry analysis of Annexin V-FITC (AV) and TO-PRO-3-iodide stained LIM2405 cells treated with 15 μM BZA alone and 15 μM BZA + 50 μM QVD in the presence of 30 ng/ml of IL-11 for 8 hours. Error bars are mean ± SEM of *n* = 3 replicates. Student’s unpaired t-test: ****p < 0.0001, **p < 0.01. **C** Z-stacked confocal microscopy images of LIM2405 cells treated with 15 μM BZA in the presence of 30 ng/ml IL-11 for 24 hours showing morphological characteristics of apoptotic cell death. **D** Z-stacked confocal microscopy images of LIM2405 cells treated with 15 μM BZA alone and 15 μM BZA + 50 μM QVD (in the presence of 30 ng/ml IL-11) for 8 hours and stained with TO-PRO-3-iodide (red) and DAPI (blue) showing early stages of apoptotic cell death. **E** Apoptotic pathway protein expression in LIM2405 cells treated with 15 μM BZA in the presence of 30 ng/ml IL-11 treatment for 24 hours. Data is the mean ± SD of *n* = 2 replicates. Statistical analysis was conducted using a two way ANOVA with Tukeys multiple comparisons test. P-values are indicated in Supplementary table 2.

Next, we sought to validate our findings in a microsatellite instable (MSI) colorectal cancer model using the LIM2405 cell line [24]. We evaluated the relative mRNA expression of *ESR1 and ESR2* in LIM2405 cells, and they were found to be minimally expressed compared to the expression of *IL11, IL11RA and IL6ST* (Fig. S4A). We confirmed that LIM2405 cells were responsive to BZA as there was a dose-dependent decrease in pSTAT3 protein expression on treatment with BZA and stimulation with IL-6 and IL-11 (Figs. 3A and S6D). BZA treatment in vitro also increased cellular apoptosis (Fig. 3B). Co-treatment with BZA, in combination with Q-VD-OPh (QVD), a pan-caspase inhibitor, prevented cell death (Fig. 3B). Flow cytometry scatter plots for each treatment are shown in Fig. S4B. Confocal imaging of BZA-treated cells showed the morphological features of apoptotic cells characterised by cell membrane blebbing and protrusions (Fig. 3C). Compared to vehicle control treated cells, increased uptake of TO-PRO-3 and DAPI stains (indicative of early-stage apoptosis) was also measured with BZA treatment (Fig. 3D). Additionally, BZA, in combination with QVD, reduced TO-PRO-3 and DAPI stain uptake (Fig. 3B, D) and reversed morphological features of apoptosis (Fig. 3D). Consistent with these observations, BZA treatment induced the upregulation of pro-apoptotic proteins of the Bcl-2 family (Bad, Bax), members of the TNF-α family (TRAIL-1, TRAIL- 2, TNF-α and CD40), p53, the IAP family of proteins (SMAC and HTRA) and heat shock proteins (HSP-27 and HSP-60) (Fig. 3E). The upregulation of these particular apoptotic markers was similar in profile to that observed in BZA-treated HT29 cells. Collectively, these results indicate that BZA treatment effectively induces tumour cell apoptosis in LIM2405 cells.

### Inhibition of gp130/STAT3 signalling in combination with SMAC-mimetics potently enhances pro-apoptotic and anti-proliferative responses in colorectal cancer cells

**Fig. 4:**
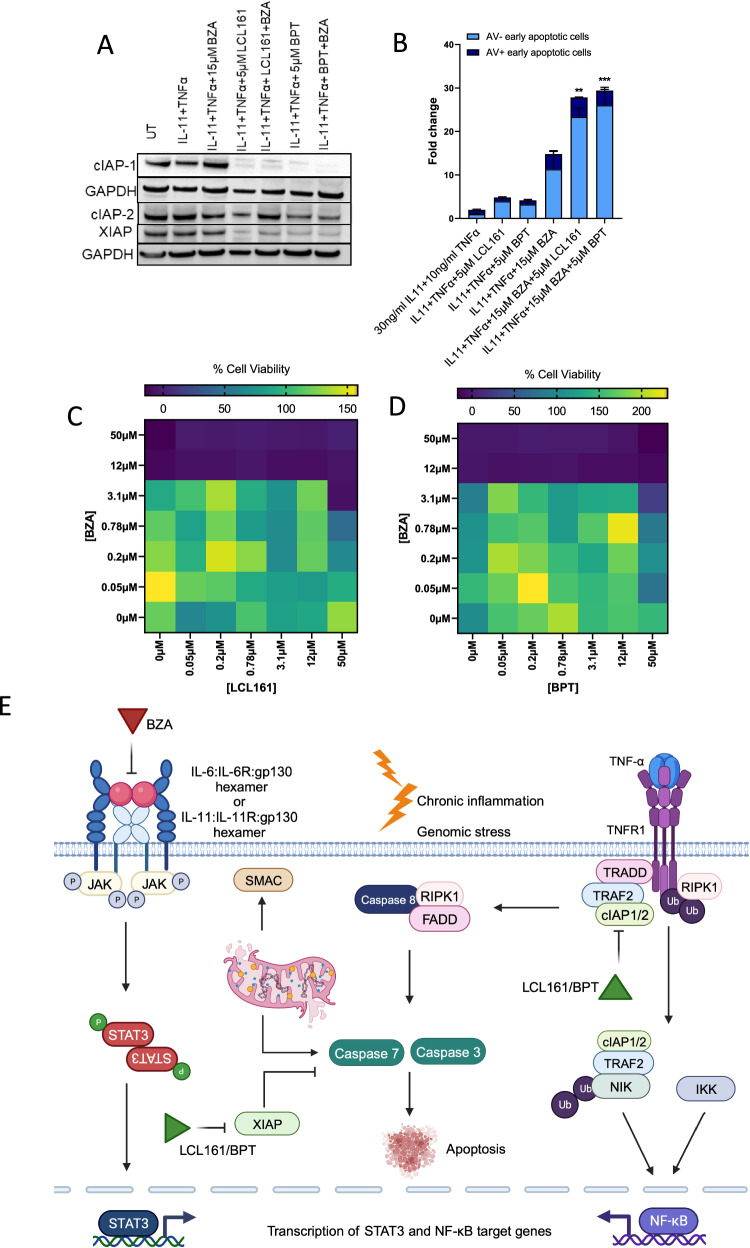
BZA-dependent inhibition of gp130/STAT3 signalling sensitises colorectal cancer cells to SMAC-mimetic-induced apoptotic cell death. **A** Western blot of cIAP-1, cIAP-2, XIAP and GAPDH protein expression in LIM2405 cells treated with 15 μM BZA, 5 μM LCL161, 5 μM BPT (Birinapant) alone and 15 μM BZA + 5 μM LCL161, 15 μM BZA + 5 μM BPT in the presence of 30 ng/ml IL-11 and 10 ng/ml TNF-α for 24 hours. **B** Flow cytometry analysis of Annexin V-FITC and TO-PRO-3-iodide stained LIM2405 cells treated with 15 μM BZA, 5 μM LCL161, 5 μM BPT (Birinapant) alone and 15 μM BZA + 5 μM LCL161, 15 μM BZA + 5 μM BPT in the presence of 30 ng/ml IL-11 and 10 ng/ml TNF-α for 8 hours. Error bars are mean ± SEM of *n* = 3 replicates. Student’s unpaired t-test: ***p < 0.001, **p < 0.01. **C**, **D** % Cell viability of LIM2405 cells treated with the indicated concentrations of (**C**) BZA + LCL161 and (**D**) BZA + BPT in the presence of 30 ng/ml IL-11 and 10 ng/ml TNF-α for 8 hours compared to the untreated control. Data is representative of the mean of *n* = 3 replicates. Statistical analysis was conducted using a two way ANOVA with Tukeys multiple comparisons test. Interaction factor between BZA and LCL161 p < 0.001. Interaction factor between BZA and BPT p < 0.05. **(E)** Schematic representation of the proposed mechanism of action of BZA combined with SMAC-mimetics induce tumour cell apoptosis.

Since BZA induces apoptosis in colorectal cancer via suppression of gp130/STAT3 inflammatory signalling, we assessed whether the combination of BZA and SMAC-mimetics could be beneficial for colorectal cancer treatment. We confirmed that LCL161 and Birinapant induced the degradation of cIAP-1 and XIAP proteins even in combination with BZA using Western blots (Figs. 4A and S6E). We then assessed whether BZA in combination with SMAC-mimetics induced cellular apoptosis by co-treating the cells with BZA and either LCL161 or Birinapant in the presence of IL-11 and TNF-α in serum-free media and observed significantly increased amounts of Annexin V and TO-PR-O3 staining in cells (Fig. 4B). Flow cytometry scatter plots for each treatment are shown in Fig. S4C. Co-treatment of these cells with BZA and either LCL161 or Birinapant also reduced cell proliferation within the first 8 hours of treatment before apoptosis affected cell viability (Fig. 4D, E). cIAP-1/2 and XIAP promote cell survival by E3-mediated ubiquitination of caspase proteins [25–27]. In a TNF-α-stimulated tumour cell in the inflammatory colorectal cancer microenvironment, upon binding of TNF-α to its receptor TNFR1, adaptor proteins TRADD and TRAF2 are recruited with cIAP-1 and/or cIAP-2 and RIP kinase [25–27] (Fig. 4E). RIP serves as the substrate that is required for cIAP-1/2 mediated ubiquitination that activates the NF-Κβ signalling pathway and also prevents the formation of the death-inducing cytosolic complex required to activate caspase 8 [25–27] (Fig. 4E). On the other hand, XIAP directly binds to caspase 3 and 7 to prevent apoptosis induction [25–27] (Fig. 4E). SMAC-mimetics LCL161 and birinapant interact with XIAP, and inhibit it’s anti-apoptotic activity, thereby promoting the activation of caspases 3 and 7 [25, 27]. They also bind to the BIR domain of cIAP-1 and cIAP-2 to induce autoubiquitination and proteasomal degradation, thereby leading to a pulse of NF-Κβ signalling and triggering the release of TNF-α, IL-6, IL-11 and gp130 [25–30]. Therefore, co-treatment of bazedoxifene with LCL161 and birinapant greatly sensitises the cancer cell to TNF-mediated apoptosis.

Collectively, our findings suggest that co-targeting gp130 in combination with SMAC-mimetics could represent a promising new therapeutic strategy in colorectal cancer. Importantly, this is the first study to identify potential cooperative effects between gp130/STAT3 signalling inhibitors and SMAC-mimetics.

### gp130/STAT3 signalling inhibition in combination with chemotherapy induces apoptosis in patient-derived colon cancer organoids

**Fig. 5:**
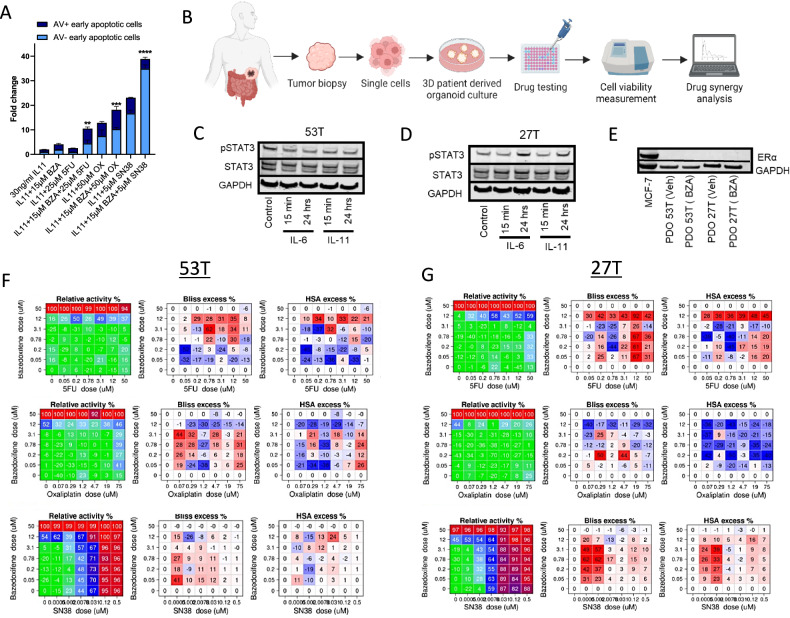
BZA-dependent inhibition of gp130/STAT3 signalling demonstrates synergistic activity with the standard-of-care chemotherapy. **A** Flow cytometry analysis of Annexin V-FITC (AV) and TO-PRO-3-iodide stained LIM2405 cells treated with 15 μM BZA, 25 μM 5-fluorouracil (5FU), 50 μM oxaliplatin (OX), 5 μM SN38 alone and in combination (BZA + 5FU, BZA + OX and BZA + SN38) in the presence of 30 ng/ml IL-11 for 24 hours. Error bars are mean ± SEM of *n* = 3 replicates. Student’s unpaired t-test: ****p < 0.0001, ***p < 0.001, **p < 0.01. **B** Schematic representation of organoid experiment workflow. Tumour biopsies were taken from the sigmoid and ascending colon of patients with stage II and stage III colon cancer, processed into single cancer cells and then cultured as 3D patient-derived organoids (PDO) to be tested using novel drug combinations. **C** Western blot of pSTAT3 and total STAT3 protein levels in PDO 53 T stimulated with IL-6 and IL-11 for 15 min and 24 hours. **D** Western blot of pSTAT3 and total STAT3 protein expression in PDO 27 T stimulated with IL-6 and IL-11 for 15 min and 24 hours. **E** Western blot of ERα protein in PDO 53 T and PDO 27 T treated with 3 μM BZA for 7 days. **F**, **G** Cell viability, Bliss excess scores and HSA excess scores of the indicated concentrations of BZA + 5FU, BZA + OX and BZA + SN38 in PDO 53 T and 27 T over 7 days. High synergy scores are represented in red and low scores indicating antagonistic effects are represented in blue.

Most patients with colorectal cancer exhibit poor responses to chemotherapy [31, 32]. Therefore, standard-of-care treatments have progressed towards combinations of chemotherapy and targeted therapies. To evaluate whether targeted gp130/STAT3 inhibition could enhance the cytotoxic effects of chemotherapy treatment, LIM2405 colorectal cancer cells were treated with BZA in combination with standard-of-care chemotherapies, including 5-fluorouracil (5-FU), oxaliplatin and SN38. All treatment combinations significantly induced apoptosis compared to single-drug treatment groups (Fig. 5A). Notably, the most potent effects were observed following co-treatment with BZA and SN38, which induced a 40-fold increase in apoptosis compared to the vehicle-treated cells (Fig. 5A). Flow cytometry scatter plots are shown in Fig. S5A.

Patient-derived organoids play a prominent role in preclinical and translational drug screening projects as they retain key genetic and phenotypic characteristics from the original tumour [33]. For this reason, they can be good predictors of drug responses. Since multi-target drug combination strategies have shown great promise in improving treatment efficacy and overcoming drug resistance, we assessed the combination of gp130 inhibition by BZA with standard-of-care chemotherapy drugs in well-characterised patient-derived colorectal cancer organoids (Fig. 5B). As previously published, organoids were generated from ascending and sigmoid colon tumours from patients with late-stage C and D disease [34, 35]. We identified the presence of an intact gp130 signalling cascade in tumour organoids obtained from colorectal cancer patients by evaluating IL-6 and IL-11-dependent phosphorylation of STAT3 (Figs. 5C, D and S6F). Although all organoids were positive for gp130 expression (Fig. S6F), patient-derived organoid 53 T did not show increased pSTAT3 levels when treated with IL-6 and IL-11 in culture (Figs. 5C and S6F).

In contrast, patient-derived organoid 27 T showed increased pSTAT3 protein levels when treated with IL-6 and IL-11 (Figs. 5D and S6F). As BZA is a SERM, we evaluated the protein expression of ERα in the organoids, using the ER-positive breast cancer cell line MCF-7 as a comparison (Figs. 5E and S6G). Both tumour organoids did not express ERα in the vehicle or BZA-treated conditions (Figs. 5E and S6G).

Next, we compared the therapeutic efficacy of oxaliplatin, 5-FU and SN38 either alone or in combination with BZA on the in vitro growth rates of organoids. While we could not detect synergistic effects between BZA and 5-FU or oxaliplatin, respectively, co-treatment with BZA and SN38 resulted in a significant antiproliferative effect in patient-derived tumour organoids (Fig. 5F-G). As predicted, more potent drug synergy was observed in patient-derived organoid 27 T (Fig. 5G), which responded to stimulation with IL-6 and IL-11 (Fig. 5D), compared to 53 T (Fig. 5C, F). This data suggests a promising role for using patient-derived organoids to predict drug responses and demonstrates that BZA effects are specific only to colon tumours showing IL-6 and IL-11-dependent STAT3 signalling. These findings suggest that combined inhibition of topoisomerase I and gp130/STAT3 could provide an effective novel treatment combination. The synergistic effect of BZA and SN38 reduces the dose required for each drug to induce apoptosis in colorectal cancer cells.

## Discussion

Aberrant STAT3 activation is observed in most colorectal cancer patients and is associated with reduced overall survival [11, 12]. For this reason, therapeutic strategies that suppress STAT3 activation represent a promising approach to developing new colorectal cancer drugs. The gp130 receptor is the most prominent upstream activator of STAT3. This study demonstrated that BZA directly binds to gp130, inhibiting IL-6 and IL-11 responses in several human colon cancer cell lines, xenograft, and patient-derived organoid models. We demonstrated the contribution of gp130 signalling to tumour growth in vivo, using human colon cancer xenografts and that the gp130 receptor specifically mediates BZA treatment-associated inhibition of human colon cancer cell proliferation. We discovered that BZA treatment enhances the pro-apoptotic effects of SMAC-mimetics LCL161 and Birinapant. We also provide the first evidence of the synergistic response of BZA with topoisomerase I inhibitor SN38 as a promising new treatment combination.

IL-6 and IL-11 are the only members of the IL-6 family of cytokines that signal via the formation of a hexameric protein complex by first binding to the non-signalling cytokine-specific receptors (IL-6R and IL-11R, respectively), followed by recruitment of two gp130 receptor molecules [36–38] (Figs. 1A, B and S1A). These cytokines are produced by many cell types in the tumour microenvironment, including stromal cells, tumour-infiltrating immune cells and the tumour cells themselves. IL-6 and IL-11 drive tumour cell proliferation, survival, invasiveness and metastasis while suppressing anti-tumour immune responses [36, 39–41]. IL-6 is a potent driver of colorectal tumorigenesis via downstream activation of gp130/STAT3 signalling, and IL-6 elevated expression levels correlate with poor patient prognosis [42]. However, treatments with tocilizumab or other monoclonal antibodies against IL-6 are associated with adverse effects such as gastrointestinal haemorrhage, thrombocytopenia, and neutropenia [6]. Meanwhile, IL-11 has emerged as an essential driver of colorectal cancer tumorigenesis [7, 43, 44]. Moreover, IL-11 secreted from cancer-associated fibroblasts in response to TGF-β activates gp130/STAT3 signalling to promote the survival of metastatic tumour cells [20]. Our observations demonstrate that dual targeting of IL-6 and IL-11 signalling represents a promising treatment strategy for colorectal cancer. Unlike IL-6 inhibitors currently undergoing clinical evaluation, BZA offers several benefits, including a convenient oral administration route, low antigenicity, and low toxicity [6]. Furthermore, BZA is FDA-approved for treating post-menopausal osteoporosis and has demonstrated a well-tolerated and favourable safety profile (NCT00205777) [45, 46]. Additionally, our studies show that BZA treatment was effective in cell lines from both MSI and MSS colorectal cancer subtypes, demonstrating the broad utility of BZA in treating colorectal cancer subtypes.

We discovered that BZA treatment increases the expression of TNF-α and the IAP family of proteins suggesting that BZA treatment in combination with SMAC-mimetics could co-operatively induce apoptosis in colorectal cancer. Indeed, BZA treatment combined with Birinapant and LCL161 potently induced apoptosis and reduced cancer cell proliferation compared to single-drug treatment effects (Fig. 4). These findings confirm that STAT3 and NF-κβ co-operatively control overlapping gene expression of anti-apoptotic and pro-proliferative genes [28]. Moreover, STAT3 is also required to maintain constitutive NF-κβ activity in tumour cells [47]. Activation of NF-κβ also induces the expression of pro-inflammatory cytokines, including TNF-α and IL-6, which are required for STAT3 activation. This suggests that the crosstalk between NF-κβ and STAT3 signalling pathways drive cancer progression [28, 29, 48, 49]. Additionally, treatment with LCL161 can induce a robust inflammatory response via activation of macrophages and dendritic cells, which triggers the release of pro-inflammatory cytokines, including TNF-α and IL-6 [27]. Treatment of *Apc*^min/+^ mice with the STAT3 inhibitor BP-1-102 [50] reduced the growth of colonic tumours via the downregulation of STAT3 activity and STAT3-dependent NF-κβ activation [51]. In addition, the sphingosine-1-phosphate inhibitor FTY720 [52] reduces colitis-associated colorectal tumour growth via indirect downregulation of STAT3 and activation of NF-κβ signalling [53]. These observations support the rationale that combined targeting of gp130/STAT3 signalling and NF-κβ signalling using BZA and SMAC-mimetics could be a novel and effective treatment strategy in colorectal cancer. Both LCL161 (NCT01240655, NCT01098838, NCT02890069) and Birinapant (NCT02587962, NCT01188499, NCT00993239, NCT01573780) were well tolerated and displayed minor toxicity profiles in phase I and II clinical trials for the treatment of solid tumours including gastrointestinal cancers [54]. In preclinical studies, Birinapant sensitised colorectal cancer cells to 5-FU, and oxaliplatin-mediated apoptosis reduced tumour growth in colorectal cancer cells xenografts [55]. Furthermore, in the phase II clinical trial, Birinapant demonstrated clinical benefit in patients who relapsed with irinotecan treatment [56].

Interestingly, we discovered that BZA treatment, in combination with the topoisomerase I inhibitor, SN38, synergistically reduced cell proliferation and induced apoptosis in colorectal cancer cells and patient-derived organoids. This suggests that BZA could be used in an adjuvant setting to improve the efficacy of standard-of-care treatments at reduced doses. Previous reports have shown that the STAT3 pathway is activated in response to topoisomerase I inhibition [57]. Hence the synergistic benefits observed with BZA and SN38 co-treatment may present a therapeutic opportunity to overcome resistance to topoisomerase I inhibitors. Additionally, combining BZA and irinotecan therapy may reduce the dosing of irinotecan and therefore reduce treatment resistance from developing in patients.

Our study provides the first preclinical evidence of BZA as a promising new treatment strategy for colorectal cancer patients where tumour progression is driven by gp130/STAT3 signalling.

## Materials and methods

### Cell culture and maintenance

Human colorectal cancer cells were obtained from the American Type Culture Collection (ATCC). The HT29 short hairpin control and short hairpin gp130 knockdown cells were kindly provided by Prof Batlle [20]. All cell lines were maintained at 37 °C in 10% CO_2_ with Dulbecco’s modified Eagle’s medium (DMEM)/F-12 supplemented with 10% v/v fetal bovine serum (Corning). HT29 cell lines (sh-co and sh-130) were supplemented with 50 µM doxycycline and 10 µM puromycin for cell growth selection. Cells were washed using PBS (Thermo Fisher), lifted off using Tryple E (Thermo Fisher) and resuspended in culture media (1:10) for maintenance.

### Drug treatments

The following drug treatments were used in the study: bazedoxifene (BZA) (Selleck Chem; S212850), 5-fluorouracil (5-FU) (Accord), oxaliplatin (OX) (Accord), SN38 (Selleck Chem; S4908), LCL161 (Selleck Chem; S7009) and Birinapant (Selleck Chem; S7015).

The following cytokine treatments were used in the study: human recombinant IL-6 (Thermo Fisher; BMS341), human recombinant IL-11 (Thermo Fisher; PHC0115) and human recombinant TNF-α (Thermo Fisher; RP75738).

### Flow cytometry

For Annexin V TO-PRO-3-iodide staining, adherent cells were treated with combinations of 15 μM BZA, 25 μM 5-FU, 50 μM OX and 5 μM SN38, and 30 ng/ml IL-11. Cells were lifted off using Tryple E (Thermo Fisher), washed twice in PBS (Thermo Fisher) and then stained with Annexin V and TO-PRO-3-iodide according to the previously published protocol by [23]. The gating strategy is shown in Fig. S2A.

For sub-G1 analysis, adherent cells were treated with combinations of 15 μM BZA, 25 μM 5-FU and 50 μM OX, and 30 ng/ml IL-11 for 72 hours. Cells were scraped in treatment media, washed twice with PBS and incubated with Propidium Iodide (50 mg/100 m of sodium citrate buffer) and RNAse (1 µg/ml) overnight at 4 °C.

### Protein array

HT29 (sh-co and sh-130) and LIM2405 cell lines were grown in 10 mm tissue culture dishes, treated with 30 ng/ml IL-11 and 15 µM BZA in serum-free culture media and then lysed using RIPA buffer containing protease inhibitor cocktail and phosphatase inhibitor cocktail. Protein lysates containing 400 μg of protein were analysed using Human Apoptosis Antibody Array-Membrane (Abcam ab134001) containing 43 target antibodies according to the manufacturer’s instructions. Targets included were Bad, Bax, Bcl-2, Bcl-1, BID, BIM, Caspase 3, Caspase 8, CD40, CD40L, cIAP-2, cytoC, DR6, Fas, FasL, HSP27, HSP60, HSP70, HTRA, IGF-I, IGF-II, IGFBP-1, IGFBP-2, IGFBP-3, IGFBP-4, IGFBP-6, IGF-1sR, livin, p21, p27, p53, SMAC, Survivin, sTNFR1, sTNFR2, TNF-α, TNF-β, TRAILR-1, TRAILR-2, TRAILR-3, TRAILR-4 and XIAP. Array membranes were detected using chemiluminescence signals on the Chemi-doc XRS+ (Bio-Rad), and signal quantification was carried out using ImageJ software.

### Gene expression analysis

RNA was isolated from cell lines, and quantitative reverse-transcriptase PCR was performed as described in [5]. Relative expression was calculated using the comparative CT (2^-ddCt^) method after normalisation to *GAPDH* as the housekeeping gene [58]. Probes used in this study include *ESR1* (#Hs01046816_m1), *ESR2* (#Hs01100353_m1), *IL6ST* (#Hs00174360_m1), *GAPDH* (#Hs02786624_g1), *IL11* (#Hs01055414_m1) and *IL11RA* (#Hs00234415_m1).

### Western blot analysis

Cells or organoids were lysed using RIPA buffer containing a complete mini protease inhibitor cocktail (Roche) and PhosphoSTOP phosphatase inhibitor cocktail (Roche). Protein was quantified using the Pierce BCA Protein Assay Kit (Thermo Fisher). Western blot analysis was performed using 30 μg of protein lysates, as described in [5]. The antibodies used are described in Table S1. We thank Prof. John Silke (WEHI) for providing the c-IAP2 antibody as a gift.

### Animal experiments

Animal experiments were conducted under ethics protocols approved by the Austin Health Animal Ethics Committee. Mice used in this experiment (male and female) were housed at a constant temperature of 24 °C with a 12 hour light/dark cycle, 4 mice per cage with food and water *ad libitum*. Mice were 10 weeks of age at the start of the experiment. Colon cancer cells suspended in Matrigel (Corning) in a 1:1 ratio were injected into the abdominal flank of each mouse subcutaneously. Palpable tumours were measured using electronic callipers (Adelab scientific) three times a week. Drug treatments began when HT29 sh-co tumour-bearing mice developed palpable tumours (average tumour size 100 mm^3^). BZA (3 mg/kg, Selleck Chem) was dissolved in 10% v/v DMSO and 90% v/v sterile sunflower oil and administered via i.p injection 5 times a week for three consecutive weeks. Vehicle-treated cohorts received an i.p injection of 10% v/v DMSO and 90% v/v sunflower oil at the same frequency and duration as BZA-treated mice. Mice were euthanised by CO_2,_ and tumour tissue was collected and snap-frozen for further analysis.

### Differential scanning fluorimetry (DSF)

Human gp130 D1-D6 protein was expressed and purified by GeneScript, USA. 400 μg of the gp130 protein was incubated with 2.5 µM BZA at RT for 5 min and analysed for thermal unfolding using the Tycho NT.6.

### Microscale thermophoresis (MST)

The MST analyses were performed using a Monolith NT.115 (NanoTemper Technologies). Recombinant gp130 ECD protein (i.e. gp130 D1-D6) was labelled using the Monolith Labelling kit RED-NHS 2^nd^ Generation (Nanotemper Technologies) following the manufacturer’s protocol. BZA was titrated from 250 µM in a 1:1 series of sixteen dilutions in PBS 0.05% Tween20. Each dilution of BZA was mixed with an equal volume of the labelled 80 nM gp130 ECD. Samples were loaded into Monolith Standard capillaries. The MST LED power was set to medium with 40% excitation power at RT. MST control and data analysis was carried out using MO. Control (v1.6) and MO. Affinity Analysis (v2.3) software.

### Structural modelling

The model of the IL-6 receptor ECD was constructed by aligning the full ECDs of IL-6R (PDB ID: 1N26) [59] and gp130 (AlphaFold2 model, https://alphafold.ebi.ac.uk/entry/P40189) to the corresponding partial structures within the hexameric IL-6:IL-6R:gp130 crystal complex (PDB ID: 1P9M) [60] using PyMOL 2.1.1 (The PyMOL Molecular Graphics System, Version 2.0 Schrödinger, LLC). The hexameric signalling complex is comprised of two IL-6:IL-6R:gp130 trimers; in Fig. 1A, an asterisk denotes the components of one trimer and only the ECD of IL-6R and gp130 have been modelled. The individual proteins are depicted as molecular surfaces. The three ECD subunits of IL-6R and six ECD subunits of gp130 are labelled in Fig. 1A; the N-terminal domain for each protein is D1. The flexible juxtamembrane regions (JM) of IL-6R and gp130, and the membrane surface, are represented schematically in the figure. Two orthogonal views are shown in Fig. 1A; the left image is a side view, showing the relative position of the cell membrane, and the right image is the view looking down onto the membrane surface. IL-6 interacts with IL-6R via Site I and with gp130 via Sites II and III, the location of these interfaces is indicated in the right image. The model of the IL-11 receptor ECD was constructed from the IL-6 receptor ECD model by aligning IL-11 (PDB ID: 6O4O) [61] with IL-6 and IL-11R (PDB ID: 6O4P) [61] with IL-6R. In silico docking of BZA to the surface of gp130 D1 was manually carried out using SYBYL-X 2.1.1 (Certara, L.P. Princeton, NJ, USA) as previously described [5].

### STAT3 activity assay

Transfections were performed in HEK293 cells using Lipofectamine 2000 (Invitrogen) according to the manufacturer’s instructions. Cells were seeded into 6-well plates and transfected with 100 ng of p-APRE luciferase, 100 ng of pRL-TK-Renilla Luciferase expression constructs, 100 ng of pc-DNA hIL-6R/hIL-11R (source) in a 3:1 Lipofectamine: DNA ratio [5]. After serum starvation for 24 hours, cells were treated with 30 ng/ml IL-6/IL-11 and 10 µM BZA (Selleck Chem). Luciferase assays were performed using the Dual Luciferase Reporter Assay Kit (Promega) according to the manufacturer’s instructions. Firefly luciferase assays were normalised to Renilla activity.

### Proliferation assay

HT29 sh-co and sh-130 cells in culture media solution were first plated in 96 well plates at plating density and proliferative activity was measured using the Real Time-Glo MT cell viability assay (Promega) over the course of 3 days according to the manufacturer’s instructions.

### Confocal microscopy

Cells in culture media were plated in 4-well chambered slides (Labtek-II), serum starved for 24 hours and then treated with 15 µM BZA for a further 24 hours. Cells were then incubated with 1 µM TO-PRO-3-iodide (Life Technologies) and 1 µM DAPI (Sigma-Aldrich) diluted in PBS for 15 min at RT. Fluorescence readouts were imaged using Zeiss Confocal LSM 980 at the ACRF Centre for Imaging the Tumour Microenvironment. Images were processed using Zeiss Zen (Blue edition).

### Patient-derived organoid cultures

Patient-derived organoids were obtained from resected tumours of patients with colorectal cancer. All patients provided informed consent before tumour collection, and the study was approved by the Human Research Ethics Committee (HREC 2016.249). Organoids were grown in DMEM/F-12 media with Matrigel (Corning) and seeded in 384 well plates [34]. After 72 hours of establishment, the organoids were treated with equimolar concentrations of BZA, 5-FU, OX and SN-38 (50 μM - 0 μM) for 7 days. Cell viability was determined using CellTitre-Glo 3D (Promega) according to the manufacturer’s instructions using the EnVision Plate Reader (PerkinElmer). Fluorescence readouts were converted into relative activity using DMSO as a negative control (vehicle treatment) and 1 μM bortezomib as a positive killing control.

### Drug synergy analysis

Drug synergy was calculated using two models: the Highest Single Agent (HSA) excess model, which assumes that the expected effect of a drug combination is higher than the effect of a single drug at its highest combination [62], and the Bliss independence model, which assumes statistic independence between two compounds by assuming that two drugs target independent biological signalling pathways [63]. Synergy scores less than -10 indicate that the interaction between the two drugs is antagonistic, scores between -10 and 10 suggest that the interaction is additive, and scores higher than 10 indicate synergistic combinations. All calculations and specific functions were performed in R Version 3.0.1.

### Statistical analysis

All data were analysed using GraphPad PRISM statistical software as indicated in the figure legend. A *P*-value of 0.05 were considered significant.

## Supplementary information

Supplementary material (text and figures)

Supplementary Table 2

Checklist

## Data Availability

All data associated with this study are present in the paper or supplementary materials. Materials can be requested by emailing Ashwini.chand@onjcri.org.au.
