# Correction: Combination of bazedoxifene with chemotherapy and SMAC-mimetics for the treatment of colorectal cancer

**DOI:** 10.1038/s41419-025-07631-y

**Published:** 2025-05-12

**Authors:** Rhynelle S. Dmello, Michelle Palmieri, Pathum S. Thilakasiri, Larissa Doughty, Tracy L. Nero, Ashleigh R. Poh, Sarah Q. To, Erinna F. Lee, W. Douglas Fairlie, Lisa Mielke, Michael W. Parker, Ivan K. H. Poon, Eduard Batlle, Matthias Ernst, Ashwini L. Chand

**Affiliations:** 1 Olivia Newton-John Cancer Research Institute and School of Cancer Medicine, La Trobe University, Heidelberg, VIC 3084 Australia; 2 Walter and Eliza Hall Institute of Medical Research (WEHI), Parkville, VIC 3010 Australia; 3 Department of Biochemistry and Pharmacology, and ACRF Facility for Innovative Cancer Drug Discovery, Bio21 Molecular Science and Biotechnology Institute, The University of Melbourne, Parkville, VIC 3010 Australia; 4 Department of Biochemistry and Chemistry, School of Agriculture, Biomedicine and Environment, La Trobe Institute for Molecular Science, La Trobe University, Bundoora, VIC 3083 Australia; 5 ACRF Rational Drug Discovery Centre, St. Vincent’s Institute of Medical Research, Fitzroy, VIC 3065 Australia; 6 Institute for Research in Biomedicine (IRB Barcelona), The Barcelona Institute of Science and Technology (BIST), Barcelona, Spain; 7 Centro de Investigación Biomédica en Red de Cáncer (CIBERONC), Barcelona, Spain; 8 Institució Catalana de Recerca i Estudis Avançats (ICREA), Barcelona, Spain

**Keywords:** Targeted therapies, Apoptosis, Colorectal cancer, Chemotherapy

Correction to: *Cell Death and Disease* 10.1038/s41419-024-06631-8, published online 10 April 2024

In this article, the affiliation for author Eduard Batlle is incomplete:

^6^Institute for Research in Biomedicine (IRB Barcelona), Barcelona Institute of Science and Technology (BIST), 08028 Barcelona, Spain.

It should read:

^6^Institute for Research in Biomedicine (IRB Barcelona), The Barcelona Institute of Science and Technology (BIST), Barcelona, Spain.

^7^Centro de Investigación Biomédica en Red de Cáncer (CIBERONC), Barcelona, Spain.

^8^Institució Catalana de Recerca i Estudis Avançats (ICREA), Barcelona, Spain.

The original article has been corrected.

