# Supplementary material for: Combination of bazedoxifene with chemotherapy and SMAC-mimetics for the treatment of colorectal cancer
Source: Cell Death Dis. 2024 Apr 10;15(4):255. doi: 10.1038/s41419-024-06631-8 (PMC11006905; doi:10.1038/s41419-024-06631-8)
Supplement: Supplementary file 1 — Supplementary material (text and figures) [file 41419_2024_6631_MOESM1_ESM.pdf]

List of Supplementary Materials

Supplementary Figure 1: Stepwise assembly of the IL-11 receptors and BZA putative mode of action

(A) IL-11 initially binds to IL-11R via Site I to form the binary complex (location of Sites I, II and II are shown in Figure 1B), followed by recruitment of gp130 via interaction at Site II to form the non-signalling, trimeric IL-11:IL-11Rα:gp130 receptor complex. Two trimers come together via Site III to form the hexameric signalling complex. Proteins are coloured as in Figure 1B. The IL-11R and gp130 juxtamembrane (JM) region, intracellular domain (ICD) and transmembrane helix (rectangle embedded in the membrane) have been depicted schematically, along with the location of the cell membrane. An asterisk has been used to identify protein components within a single trimer. The IL-6 receptor is thought to form in a similar stepwise manner. BZA is thought to bind to the surface of gp130 Site III (located on gp130 D1), before or after the formation of the trimeric non-signalling complex. Blockade of gp130 Site III with BZA (light pink molecular surface) prevents the formation of the hexameric signalling complex for IL-11 (or similarly IL-6).

(B) Western blot of human gp130 D1-D6 (reducing conditions) used in Figure 1.

(C-D) Luciferase reporter activity of HEK293 cells transfected with IL-6R **(C)** and IL-11R expression vectors **(D)**. Transfected cells were treated with 30 ng of recombinant cytokine alone and co-treatment with 10 µM BZA**.** Error bars are mean ± SEM of n = 3 independent experiments. One-way ANOVA with Tukey's multiple comparisons tests: ****$p \leq 0.0001$.

Supplementary Figure 2: BZA treatment reduces tumour burden in HT29 xenografts *in vivo* and induces apoptosis in HT29 cells *in vitro*

(A) Basal mRNA expression (∆Ct values) of *IL11, IL11RA, ESR1 and ESR2* in HT29 sh-co and HT29 sh-gp130 cells compared to the housekeeping gene *GAPDH*. Error bars are mean ± SEM ****p<0.0001, ***p<0.001, **p<0.01.

(B-C) Tumour volumes of HT29 sh-co **(B)** and HT29 sh-gp130 **(C)** xenograft tumours in individual mice (n = 5 per group) treated with 3 mg/kg of BZA over 14 days, as shown in Figure 2G Treatment was initiated in all experimental cohorts when HT29 sh-co xenografts reached 100mm^3 (shown as Day 1 in the graph).

(D) Flow cytometry scatter plots of HT29 sh-co and HT29 sh-gp130 cells treated with vehicle (30 ng/ml IL-11) and BZA (IL-11+ 15 µM BZA) treatments as shown in Figure 2I. The data shown is a representative sample of n = 2 independent experiments.

Supplementary Figure 3: Identification of particles in different stages of cell death using Annexin V and TO-PRO-3 staining

(A) Flow cytometry analysis showing the electronic gating strategy used to identify viable cells, AV- early apoptotic cells, AV+ early apoptotic cells and necrotic cells as previously published (23).

Supplementary Figure 4: BZA alone and in combination with SMAC-mimetics induces apoptosis in LIM2405 cells

(A) Basal mRNA expression (∆Ct values) of *ESR1* and *ESR2* in LIM2405 cells compared to expression of the house keeping gene *GAPDH*. Error bars mean ± SEM of n = 3 replicates. Students unpaired t-test: **** p<0.0001

(B) Flow cytometry scatter plots of LIM2405 cells treated with vehicle (30 ng/ml IL-11), BZA (IL-11+ 15 µM BZA) and BZA+QVD (IL-11+15 µM BZA+50 µM QVD) treatments as shown in Figure 3B. The data shown is a representative sample of n = 3 replicates.

(C) Flow cytometry scatter plots of LIM2405 cells treated with vehicle (30 ng/ml IL-11+10 ng/ml TNF-α), BZA (IL-11+TNF-α+15 µM BZA), LCL-161 (IL-11+TNF-α+5 µM LCL161), BZA+LCL161 (IL-11+TNF-α+BZA+LCL161), Birinapant (IL-11+TNF-α+5 µM BPT) and BZA+Birinapant (IL-11+TNF-α+BZA+BPT) treatments, as shown in Figure 4B. The data displayed is a representative sample of n = 3 replicates.

Supplementary Figure 5: Combination of BZA with standard-of-care chemotherapy induces apoptosis in LIM2405 cells

(A) Flow cytometry scatter plots of LIM2405 cells treated with vehicle (30 ng/ml IL-11), BZA (IL-11+ 15 µM BZA), 5-FU (IL-11+25 µM 5-FU), BZA+5-FU (IL-11+BZA+5-FU), Oxaliplatin (IL-11+50 µM OX), BZA+ Oxaliplatin (IL-11+BZA+OX), SN38 (IL-11+5 µM SN38), BZA+SN38 (IL-11+BZA+SN38). The data shown is a representative sample of n = 3 replicates.

(B) Cell cycle analysis (Sub-G1 assay) of LIM2405 cells treated with 30 ng/ml IL-11 alone and co-treated with 15 µM BZA, 50 µM oxaliplatin (OX), 25 µM 5-Florouracil (5-

FU), 15 μM BZA+50 μM OX and 15 μM BZA+25 μM 5-FU followed by Propidium Iodide (PI)

staining. Error bars are mean ± SD of n = 3 replicates. Student's unpaired t-test: ***p<0.001, ** p<0.01.

Supplementary Figure 6: Uncropped Western blots

(A) Western blot analysis of gp130 and pSTAT3 expression in HT29 sh-co (short hairpin control) and HT29 sh-gp130 (short hairpin gp130 knockdown) cells following stimulation with 30 ng/ml of IL-6 (shown in Figure 2D).

(B) Western blot of pSTAT3 and GAPDH protein expression in HT29 sh-co (short hairpin control) cells treated with 15 µM BZA for 30 min and stimulated with 30 ng/ml of IL-6 and IL-11 for 15 min as shown in Figure 2E.

(C) Western blot of Cleaved caspase 3 and GAPDH protein expression in HT29 sh-co (short hairpin control) cells treated with 15 µM BZA in the presence of 30 ng/ml IL-11 for 48 hours as shown in Figure 2K.

(D) Western blot of pSTAT3 and GAPDH protein expression in LIM2405 cells treated with BZA (3 nM - 30 µM) for 30 min and stimulated with 30 ng/ml IL-6 and IL-11 for 15 min as shown in Figure 3A.

(E) Western blot of cIAP-1, cIAP-2, XIAP and GAPDH protein expression in LIM2405 cells treated with 15 µM BZA, 5 µM LCL161, BZA+LCL161, 5 µM BPT (Birinapant)

and BZA+BPT in the presence of 30 ng/ml IL-11+10 ng/ml TNF-α for 24 hours as shown in Figure 4A.

(F) Western blot of pSTAT3 and gp130 protein expression in PDO27T and PDO53T stimulated with 30 ng/ml IL-6 and IL-11 for 15 min and 24 hours as shown in Figure 5C and Figure 5 D, respectively.

(G) Western blot of ERα protein expression in MCF-7 cells (positive control) and PDO53T and PDO27T treated with vehicle treatment control and 3 μM BZA for 7 days as shown in Figure 5E.

(H) Western blot of cleaved caspase 3 and β-actin protein expression in LIM2405 cells treated with 15 μM BZA, 25 μM 5-FU, BZA+5-FU, 50 μM OX and BZA+OX for 48 hours in the presence of 30 ng/ml IL-11 as shown in Figure S5C.

**Antibody**	**Company**	**Catalogue #**	**Clone**
pSTAT3 (Tyr705)	Cell signalling	#9145	D3A7
STAT3	Cell signalling	#4904	79D7
GAPDH	Sigma	#G8795	GAPDH-71.1
ERα	SantaCruz Biotechnology	#sc-8002	F-10
gp130	Cell signalling	#3732	polyclonal
XIAP	MBL International	#M044-3	2F1
c-IAP1	AB Clonal	#A19688	ARC0168
c-IAP2	Made in-house at WEHI	N/A	N/A

Supplementary Table 1: Antibodies used in Western blot analysis

Supplementary Table 2: Provided as an Excel file.

Figure S1

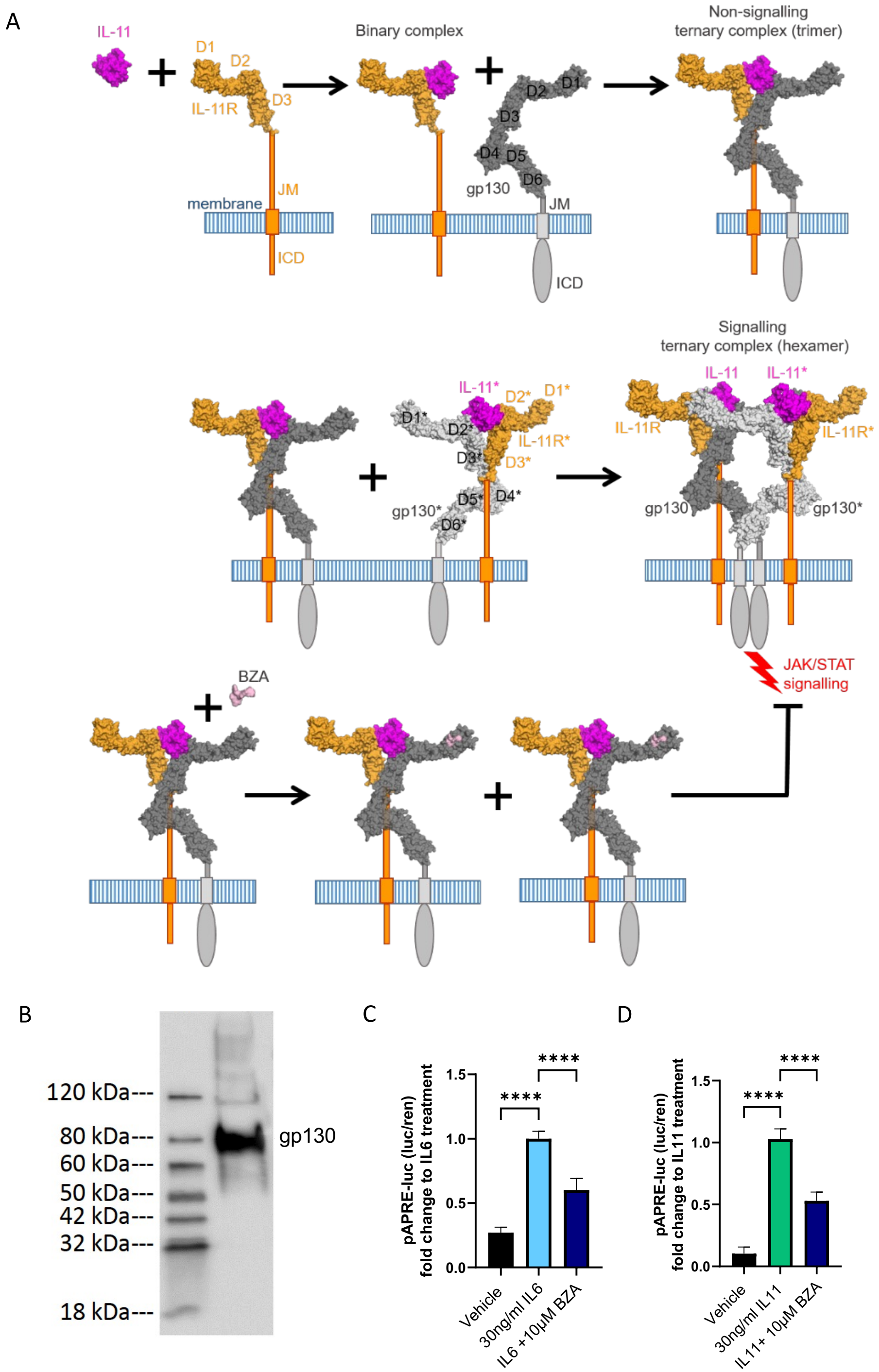
A
IL-11
D1
D2
D3
IL-11R
JM
membrane
ICD
Binary complex
D1
D2
D3
D4
D5
D6
gp130
JM
ICD
Non-signalling
ternary complex (trimer)
Signalling
ternary complex (hexamer)
IL-11*
D2*
D1*
D1*
D2*
IL-11R*
D3*
D3*
D4*
D5*
D6*
gp130*
IL-11
IL-11*
IL-11R
IL-11R*
gp130
gp130*
JAK/STAT
signalling
BZA
B
120 kDa---
80 kDa---
60 kDa---
50 kDa---
42 kDa---
32 kDa---
18 kDa---
gp130
C
pAPRE-luc (luc/ren)
fold change to IL6 treatment
****
****
Vehicle
30ng/ml IL6
IL6 +10μM BZA
D
pAPRE-luc (luc/ren)
fold change to IL11 treatment
****
****
Vehicle
30ng/ml IL11
IL11+ 10μM BZA

Figure S2

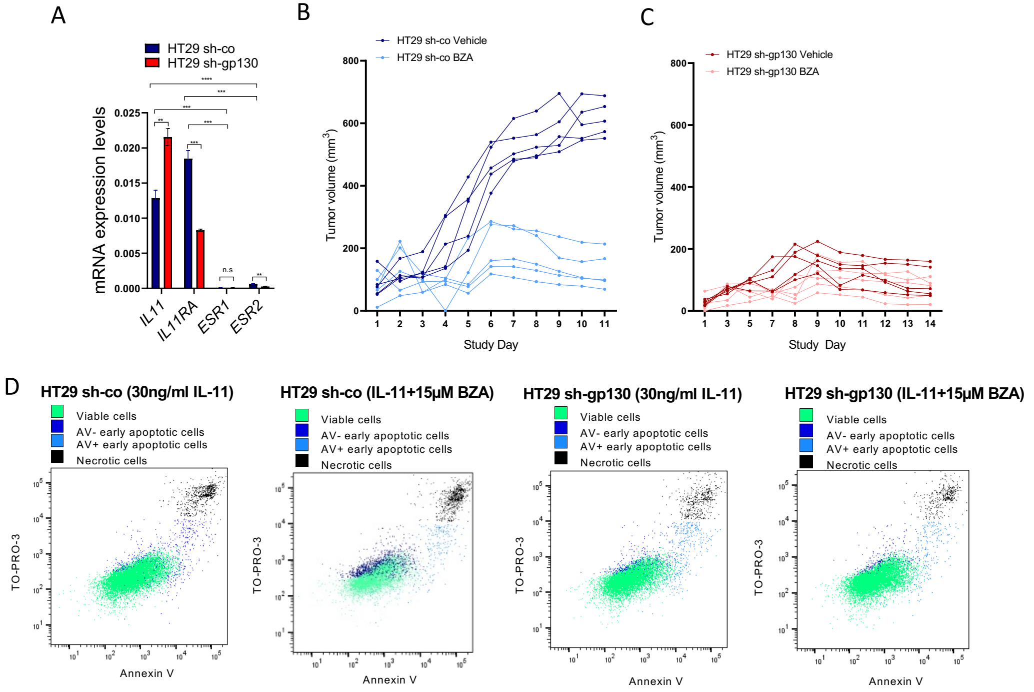
A
HT29 sh-co
HT29 sh-gp130
mRNA expression levels
IL11
IL11RA
ESR1
ESR2
n.s
B
HT29 sh-co Vehicle
HT29 sh-co BZA
Tumor volume (mm3)
Study Day
C
HT29 sh-gp130 Vehicle
HT29 sh-gp130 BZA
Tumor volume (mm3)
Study Day
D
HT29 sh-co (30ng/ml IL-11)
HT29 sh-co (IL-11+15µM BZA)
HT29 sh-gp130 (30ng/ml IL-11)
HT29 sh-gp130 (IL-11+15µM BZA)
Viable cells
AV- early apoptotic cells
AV+ early apoptotic cells
Necrotic cells
TO-PRO-3
Annexin V

Figure S3

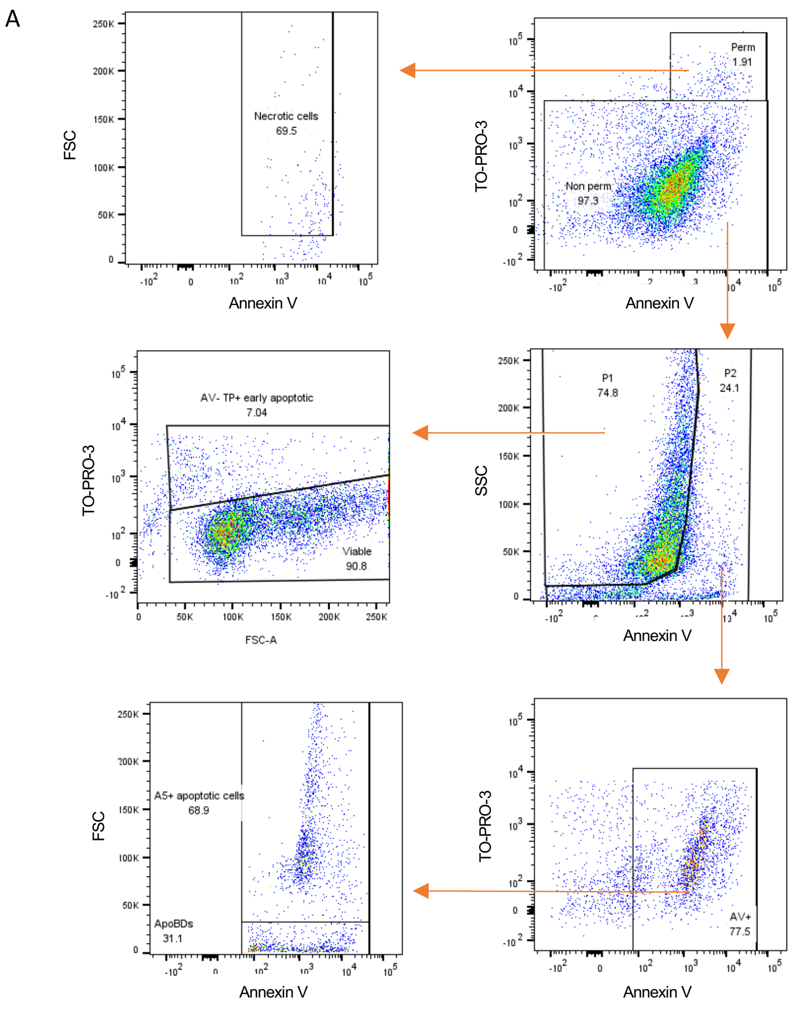
A
Necrotic cells
69.5
FSC
Annexin V
Perm
1.91
Non perm
97.3
TO-PRO-3
Annexin V
AV- TP+ early apoptotic
7.04
Viable
90.8
TO-PRO-3
FSC-A
P1
74.8
P2
24.1
SSC
Annexin V
A5+ apoptotic cells
68.9
ApoBDs
31.1
FSC
Annexin V
AV+
77.5
TO-PRO-3
Annexin V

Figure S4

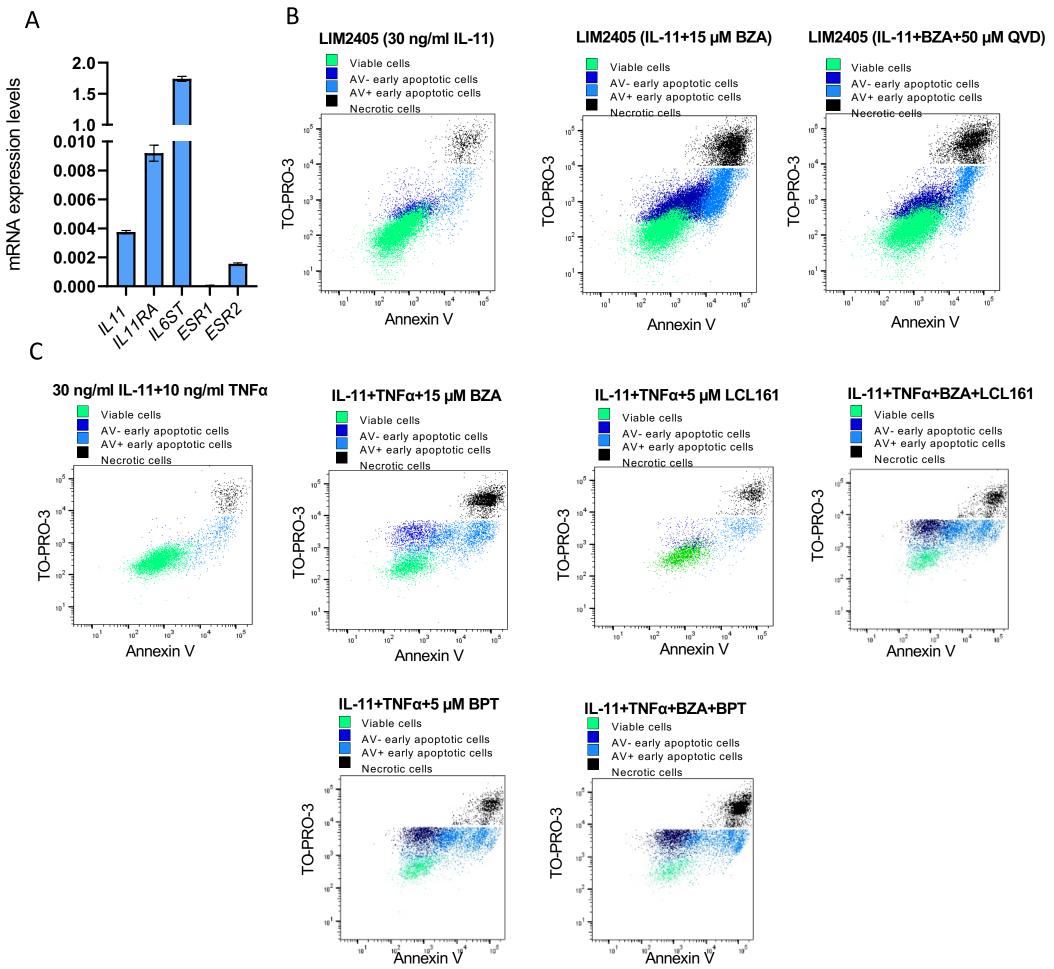
A
mRNA expression levels
2.0
1.5
1.0
0.010
0.008
0.006
0.004
0.002
0.000
IL11
IL11RA
IL6ST
ESR1
ESR2
B
LIM2405 (30 ng/ml IL-11)
LIM2405 (IL-11+15 µM BZA)
LIM2405 (IL-11+BZA+50 µM QVD)
Viable cells
AV- early apoptotic cells
AV+ early apoptotic cells
Necrotic cells
TO-PRO-3
Annexin V
C
30 ng/ml IL-11+10 ng/ml TNFα
IL-11+TNFα+15 µM BZA
IL-11+TNFα+5 µM LCL161
IL-11+TNFα+BZA+LCL161
IL-11+TNFα+5 µM BPT
IL-11+TNFα+BZA+BPT

Figure S5

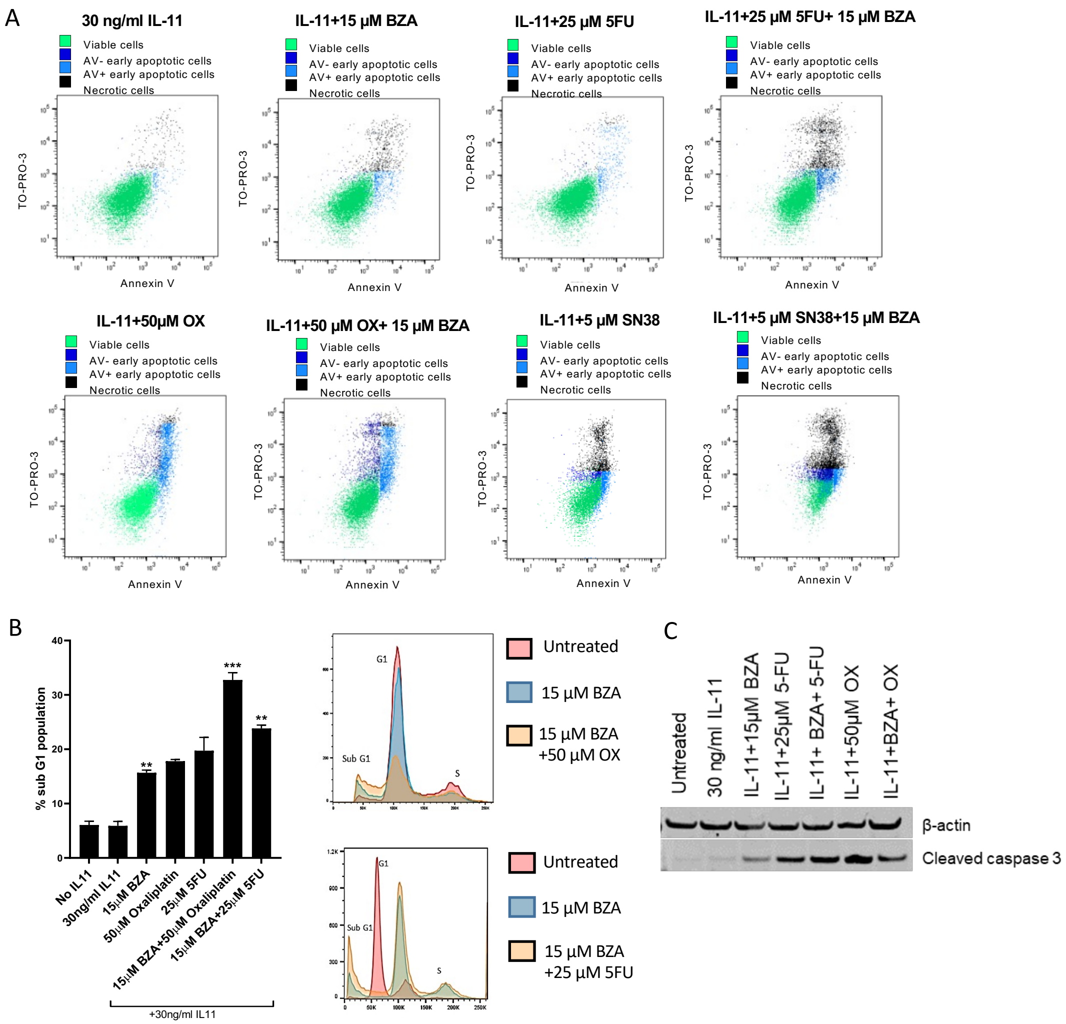
A
30 ng/ml IL-11
IL-11+15 μM BZA
IL-11+25 μM 5FU
IL-11+25 μM 5FU+ 15 μM BZA
IL-11+50μM OX
IL-11+50 μM OX+ 15 μM BZA
IL-11+5 μM SN38
IL-11+5 μM SN38+15 μM BZA
Viable cells
AV- early apoptotic cells
AV+ early apoptotic cells
Necrotic cells
TO-PRO-3
Annexin V
B
% sub G1 population
No IL11
30ng/ml IL11
15μM BZA
50μM Oxaliplatin
25μM 5FU
15μM BZA+50μM Oxaliplatin
15μM BZA+25μM 5FU
+30ng/ml IL11
G1
Sub G1
S
Untreated
15 μM BZA
15 μM BZA +50 μM OX
15 μM BZA +25 μM 5FU
C
Untreated
30 ng/ml IL-11
IL-11+15μM BZA
IL-11+25μM 5-FU
IL-11+ BZA+ 5-FU
IL-11+50μM OX
IL-11+BZA+ OX
β-actin
Cleaved caspase 3

Figure S6

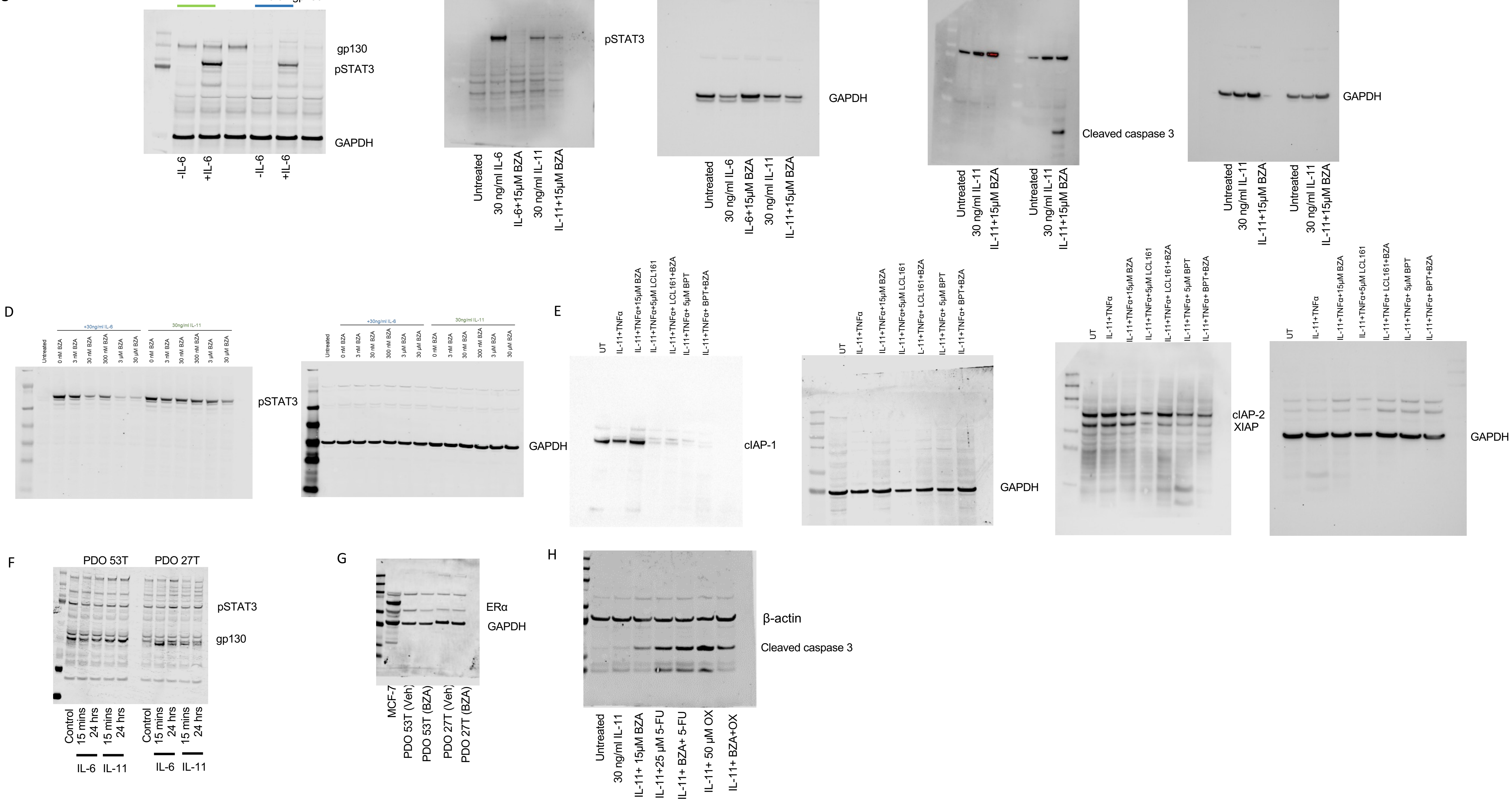

A
HT29 sh-co
HT29 sh-gp130
gp130
pSTAT3
GAPDH
-IL-6
+IL-6
-IL-6
+IL-6
B
pSTAT3
GAPDH
Untreated
30 ng/ml IL-6
IL-6+15μM BZA
30 ng/ml IL-11
IL-11+15μM BZA
C
24h
48h
Cleaved caspase 3
GAPDH
Untreated
30 ng/ml IL-11
IL-11+15μM BZA
D
+30ng/ml IL-6
30ng/ml IL-11
Untreated
0 nM BZA
3 nM BZA
30 nM BZA
300 nM BZA
3 μM BZA
30 μM BZA
pSTAT3
GAPDH
E
UT
IL-11+TNFα
IL-11+TNFα+15μM BZA
IL-11+TNFα+5μM LCL161
IL-11+TNFα+ LCL161+BZA
IL-11+TNFα+ 5μM BPT
IL-11+TNFα+ BPT+BZA
cIAP-1
GAPDH
cIAP-2
XIAP
F
PDO 53T
PDO 27T
pSTAT3
gp130
Control
15 mins
24 hrs
IL-6
IL-11
G
ERα
GAPDH
MCF-7
PDO 53T (Veh)
PDO 53T (BZA)
PDO 27T (Veh)
PDO 27T (BZA)
H
β-actin
Cleaved caspase 3
Untreated
30 ng/ml IL-11
IL-11+ 15μM BZA
IL-11+25 μM 5-FU
IL-11+ BZA+ 5-FU
IL-11+ 50 μM OX
IL-11+ BZA+OX
